# Supplementary material for: A hybrid particle-ensemble Kalman filter for problems with medium nonlinearity
Source: PLoS One. 2021 Mar 11;16(3):e0248266. doi: 10.1371/journal.pone.0248266 (PMC7951907; doi:10.1371/journal.pone.0248266)
Supplement: S1 Appendix — (PDF) [file pone.0248266.s001.pdf]

S1 Appendix Let $c_i(p)$ denote the observed mean CRPS for trial i with parameters p. It is reasonable to expect that the mean CRPS is a continuous latent function f of the filter parameters for fixed values of observed data, initial ensembles, random resamplings, and random rotations. But since these fixed values all vary in practice, we can view each f as a realization of a random field F. In this view, the quantities $c_i(p)$ are noisy observations of the random field's true mean $\overline{F}$. Our Bayesian optimizer seeks the minimizer of $\overline{F}$ using these noisy observations.

Let $\bar{c}_p$ be the mean CRPS observed over all assimilation trials that were run with parameters p. Then let $\sigma_{\bar{c}_p}$ be the empirical standard error of that mean, computed as the sample standard deviation divided by the square root of the number of trials. For convenience in setting hyperparameters of the Gaussian process model, we scale the search space to the unit cube and standardize the observations. The raw search spaces are hyperrectangles, so they are scaled in each coordinate in the obvious manner to arrive at a unit cube. To standardize the observations, we subtract the mean of the set $\{\bar{c}_p\}$, for all parameter sets p previously evaluated in the experiment, and divide the result by the sample standard deviation $\sigma_{\bar{c}_p}$ of the same set. The raw standard errors $\sigma_{\bar{c}_p}$ are simultaneously divided by $\sigma_{\bar{c}_p}$ to preserve their validity in this standardized output space. We do not introduce new notation for these transformed quantities; the remainder of this section will treat c in the standardized output space and will treat values of p in the scaled parameter space.

In these scaled spaces, we form a surrogate model supposing that f_p depends on p as a Gaussian process

$$\mathcal{GP} \sim \mathcal{N}(0, k(p, p')). \tag{16}$$

We take $k(p, p')$ to be the Matérn covariance kernel

$$k(p_i, p_j) = \frac{\Theta_s 2^{1-\nu}}{\Gamma(\nu)} \left(\sqrt{2\nu} d(p_i, p_j)\right) K_\nu \left(\sqrt{2\nu} \cdot d(p_i, p_j)\right), \tag{17}$$

where K_ν is the modified Bessel function of the second kind, and

$$d(\mathbf{p}_i, \mathbf{p}_j) = (\mathbf{p}_i - \mathbf{p}_j)^\top \mathbf{\Theta}_d^{-1} (\mathbf{p}_i - \mathbf{p}_j) \tag{18}$$

Here $\mathbf{\Theta}_d$ is a diagonal matrix of length scale hyperparameters. Each of the scalars on the diagonal of $\mathbf{\Theta}_d$ corresponds to a length scale of a feature in the space of scaled filter parameters, and each is endowed with a Gamma distribution prior $\Gamma(\lambda_L, r_L)$ with shape $\lambda_L = 6$ and rate $r_L = 3$. The factor Θ_s is another hyperparameter that controls the covariance function's overall scale, on which we also impose a Gamma distribution prior $\Gamma(\lambda_S, r_S)$ with shape $\lambda_S = 2$ and rate $r_S = 0.15$. We let the smoothness parameter $\nu = 5/2$ so that realizations are almost surely twice-differentiable. Marginalizing over the latent function f yields the posterior distribution with log density

$$\begin{aligned} \ln P\left(\mathbf{f} | \{\mathbf{p}_i\}, \Theta\right) = &- \frac{1}{2} \mathbf{s}^\top (\mathbf{K} + \mathbf{\Xi})^{-1} \mathbf{s} - \frac{1}{2} \ln |\mathbf{K} + \mathbf{\Xi}| - \frac{N_p}{2} \ln(2\pi) \\ &+ \sum_{j=1}^{N_p} \left[(\lambda_L - 1) \ln\left(\Theta_{L,j}\right) - r_L \Theta_{L,j} + \lambda_L \ln r_L - \ln \Gamma(\lambda_L)\right] \\ &+ (\lambda_S - 1) \ln\left(\Theta_S\right) - r_S \Theta_{S,j} + \lambda_S \ln r_S - \ln \Gamma(\lambda_S), \end{aligned} \tag{19}$$

where $K_{ij} = k(p_i, p_j)$ is a covariance matrix. The equation above obtains by adding the log-likelihood of our hyperparameter priors to Equation 2.30 of [1]. The GP surrogate is then fit to the rescaled data by maximizing the log-density Eq 19 using many restarts of the L-BFGS-B method [2] to arrive at the maximum a posteriori (MAP) estimator. Finally, a batch of candidate arms is generated that approximately optimizes the batched noisy expected improvement acquisition function [3] on the MAP estimator. Batch sizes varied between 1 and 32 depending on computational resources available at the time.

References

1. Williams CK, Rasmussen CE. Gaussian processes for machine learning. vol. 2. MIT press Cambridge, MA; 2006.

2. Byrd RH, Lu P, Nocedal J, Zhu C. A limited memory algorithm for bound constrained optimization. SIAM J Sci Comput. 1995;16(5):1190–1208.

3. Letham B, Karrer B, Ottoni G, Bakshy E, et al. Constrained Bayesian optimization with noisy experiments. Bayesian Analysis. 2019;14(2):495–519.
